# The effect of different whitening mouthwashes and simulated tooth brushing on surface roughness and microhardness of nanohybrid resin composite: an in vitro study

**DOI:** 10.1186/s12903-025-06002-5

**Published:** 2025-04-24

**Authors:** Cemile Yılmaz, Latife Altınok Uygun

**Affiliations:** Department of Restorative Dentistry, Faculty of Dentistry, Afyonkarahisar Health Sciences University, Afyonkarahisar, Turkey

**Keywords:** Activated charcoal, Microhardness, Nanohybrid resin composite, Patent blue V, Surface roughness, Whitening mouthwashes

## Abstract

**Background:**

This study investigated the effects of whitening mouthwashes containing active ingredients on a nanohybrid resin composite’s surface roughness (Ra) and Vickers microhardness (VHN) under distinct brushing conditions.

**Methods:**

Fifty-four disc-shaped specimens (10 mm diameter × 2 mm thickness) were fabricated and randomly assigned to three experimental conditions (*n* = 18): distilled water (Control), Patent Blue V-containing mouthwash (Colgate Optic White, COW), and activated charcoal-containing mouthwash (Colgate Plax White + Charcoal, CPWC). Each group was subdivided into brushed and non-brushed subgroups (*n* = 9). Non-brushed specimens were immersed in 20 mL of the assigned solution at 37 °C for 6 h (T1) and 12 h (T2). Brushed specimens underwent 5 000 and 10 000 brushing cycles before immersion at T1 and T2, respectively. Surface roughness was measured using a contact profilometer, and microhardness was assessed with a Vickers hardness tester. Data were analyzed using three-way mixed ANOVA, one-way ANOVA, repeated measures ANOVA, and Bonferroni correction (α = 0.05).

**Results:**

Significant differences were observed in Ra values based on mouthwash type and brushing (*p* < 0.05), while VHN remained unaffected (*p* > 0.05). In non-brushed specimens, CPWC exhibited the highest Ra increase (31.2%, + 0.296 μm), significantly higher than Control (1.6%, + 0.015 μm, *p* = 0.001) and COW (5.9%, + 0.055 μm, *p* = 0.001). Under brushed conditions, CPWC again showed the most significant increase (33.3%,+ 0.311 μm,*p* = 0.012). Microhardness showed no statistically significant changes across time points or groups (*p* > 0.05). However, at T2, CPWC (non-brushed) presented the most significant reduction (− 3.38%, − 1.4 VHN), which was significantly lower than Control (+ 1.98%, + 0.8 VHN) and COW (+ 3.3%, + 1.3 VHN) (*p* = 0.001). A 5.33% increase (+ 2.1 VHN, *p* = 0.756) was observed in the brushed CPWC group.

**Conclusions:**

Activated charcoal-containing whitening mouthwashes significantly increased surface roughness, particularly under brushing conditions, indicating a synergistic effect of chemical and mechanical wear. Microhardness values remained stable, suggesting the preservation of the internal structure. These findings highlight the need for cautious recommendation of charcoal-based mouthwashes for patients with resin restorations due to potential surface degradation risks.

*All procedures followed were by the ethical standards of the responsible committee on human experimentation (institutional and national) and with the Helsinki Declaration of 1975*,* as revised in 2008. Informed consent was obtained from all patients to be included in the study.*

## Background

Due to their favorable mechanical and optical properties, the increasing demand for esthetic dental treatments has led to continuous development in restorative materials, particularly resin composites. Nanohybrid composites, which incorporate both micro- and nano-sized fillers, offer enhanced filler dispersion, polishability, and mechanical strength, making them suitable for both anterior and posterior restorations [1, 2].

Several factors in the oral environment can affect the color and surface quality of both teeth and restorations over time. Daily habits, such as consuming colored foods and beverages, tobacco use, and exposure to various staining agents, can initiate physicochemical changes in restorative materials. Consequently, individuals often seek cosmetic dental treatments to address these esthetic concerns. Increased surface roughness of teeth and restorations make them more prone to staining because they can efficiently adsorb chromogenic substances. Additionally, fillers’ composition and particle size significantly influence restorations’ susceptibility to surface changes and discoloration [1, 3, 4, 5]. In particular, surface roughness (Ra) plays a critical role in bacterial adhesion, plaque accumulation, and discoloration. It has been reported that Ra values exceeding 0.2 μm may facilitate biofilm retention and esthetic deterioration [6, 7, 8].

Whitening products—including over-the-counter (OTC) toothpaste and mouthwashes—are widely used to remove extrinsic stains. These formulations typically contain active agents such as hydrated silica, sodium bicarbonate, calcium carbonate, calcium pyrophosphate, blue covering, hydrogen peroxide, and activated charcoal [6, 7, 8, 9, 10, 11, 12]. Although effective in enhancing dental shade, the effects of these agents on restorative materials depend on their chemical composition, abrasiveness, and pH [13, 14, 15, 16, 17, 18]. Recently, activated charcoal/carbon has gained popularity due to its effective adsorption of stains and chromogens, particularly for extrinsic tooth discoloration. Some mouthwashes now include activated charcoal/carbon and optical brighteners such as patented Blue V, known as blue covering, commonly found in whitening toothpastes [9, 12, 19, 20]. Although several investigations have evaluated the influence of whitening toothpaste on enamel and resin-based materials, limited data exist on the impact of whitening mouthwashes—particularly those containing activated charcoal or synthetic dyes—on nanohybrid composites, especially under brushing conditions [13, 14, 15, 16, 17, 18].

The present study aimed to evaluate the effects of whitening mouthwashes containing Patent Blue V and activated charcoal on the Ra and VHN of a nanohybrid resin composite under different brushing conditions. The null hypothesis was that neither the active ingredient of the whitening mouthwash nor the presence of brushing would significantly affect the surface properties (Ra and VHN) of the tested composite material.

## Methods

Ethics committee approval was obtained from the Kütahya Health Sciences University Non-Interventional Clinical Research Ethics Committee, Kütahya, Turkey (decision no: 2024/02–02). The number of specimens for the study was determined as 54 using the G*Power 3.1.9.6 power analysis program, considering a minimum sample size with 90% statistical power, an effect size of 0.27, and a 95% confidence level [13, 21].

### Specimen preparation

Fifty-four disc-shaped specimens (diameter 10 ± 0.1 mm, thickness 2 mm) were fabricated using a nanohybrid anterior resin composite (Clearfil Majesty™ Esthetic, Kuraray Noritake Dental Inc., Japan). Polyester matrix strips and cementation glass slides (1 mm thick) were placed on both sides of the mold, and light pressure was applied to standardize surface smoothness and simulate clinical finishing. By ISO 4049:2019 guidelines [22] for Class 2 materials, both the top and bottom surfaces of each specimen were polymerized using a light-emitting diode (LED) curing device (Elipar Deep Cure-S, 3M ESPE, Germany) with a power density of 1 475 mW/cm² for 20 s per surface. After polymerization, the specimens were stored in distilled water in a dark environment at 37 °C for 24 h before finishing and polishing procedures.

All specimens were pre-finished with 1000-grit silicon carbide (SiC) abrasives for 20 s under water cooling to remove surface irregularities and standardize surface texture, as recommended by ISO 4049:2019 for peripheral finishing (Sect. 7.12.2.1). The upper surfaces were further polished using a sequential polishing system (Sof-Lex™, 3M ESPE, Germany) at a low speed of 10 000 rpm with water cooling in 10 s intervals to achieve clinically relevant surface smoothness. The properties of the materials used in the study are summarized in Table 1.

**Table 1:** Materials used in the current study

Material Trade Name And Description	Type	Ingredients	Manufacturer And Lot No
Clearfil Majesty™ Esthetic	Nanohybrid Resin Composite	Bis-Gma, Hydrophobic Aromatic Dimethacrylate, Silanated Barium Glass Filler, Silanated Silica Filler, Hydrophobic Aliphatic Dimethacrylate, dl-Camphorquinone, Prepolymerized Organic Fillers 40% by Volume Particle Sizes: 0.37 μm-1,5 μm	Lot: 210111 Kuraray Noritake Dental Inc, 1621 Sakazu, Kurashiki, Okayama, Japan
Colgate Cavity Protection Toothpaste	Toothpaste	Calcium Carbonate, Aqua, Sorbitol, Sodium Lauryl Sulfate, Hyrated Silica, Arginine, Sodium Monofluorophosphate, Aroma, Cellulose Gum, Sodium Carbonate, Benzyl Alcohol, Phosphoric Acid, Sodium Saccharin, Sodium Bicarbonate, CI 77891.	Colgate Palmolive, China
Colgate^®^ Optic White	Patent Blue V-containing whitening mouthwash and alcohol-free	Aqua, Glycerin, Propylene Glycol, Sorbitol, Tetrapotassium Pyrophosphate, Zinc Citrate, PVM/MA Copolymer, Aroma, Benzyl Alcohol, Sodium Fluoride, Sodium Saccharin, Cl 42051.	Lot: 10175054 Colgate Palmolive manufacturing Poland Sp. z o.o, Aleja Colgate 2, Swidnica 58–100, Poland
Colgate^®^ Plax Charcoal	Charcoal- containing whitening mouthwash and alcohol-free	Aqua, Glycerin, Propylene Glycol, Sorbitol, Tetrapotassium Pyrophosphate, Polysorbate 20, Tetrasodium Pyrophosphate, Zinc Citrate, PVM/MA Copolymer, Aroma, Benzyl Alcohol, Sodium Fluoride, Sodium Saccharin, Bambusa Vulgaris Shoot Extract, Charcoal Powder, Cl 15510, Cl 17200, CI 19140, CI 42051.	Lot: 10207956 Colgate Palmolive manufacturing Poland Sp. z o.o. Colgate 2 Street, 58–100 Swidnica, Poland

### Brushing cycle

Initial Ra and VHN values were recorded for all specimens. Brushing groups were subjected to a brushing protocol using an automated brushing simulator (Esetron MF-100, MOD Dental, Ankara, Turkey), a validated device for simulating clinical toothbrushing dynamics. The soft-bristled toothbrush head was programmed to perform linear reciprocating motions with a 10 mm vertical stroke length at 40 mm/s under a constant 200 g vertical load. Surface roughness measurements were performed perpendicular to the brush marks to assess the changes induced by mechanical brushing accurately.

To simulate clinical brushing conditions, a homogeneous toothpaste slurry was prepared by mixing 100 g of toothpaste (Colgate Cavity Protection Toothpaste, Colgate-Palmolive, China) with 100 mL of distilled water at a 1:1 mass-to-volume ratio. The slurry and toothbrushes were replaced every 5 000 brushing cycles. Specimens were immersed in 20 mL of the tested mouthwash for 12 h to simulate twice-daily (morning and evening) 1 min rinsing [23]. The specimens were then subjected to 5 000 or 10 000 brushing cycles, representing approximately 6 months or 1 year of toothbrushing in healthy individuals [6, 7, 14]. Subsequently, the synergistic effects of 1 year of simulated in vivo toothbrushing combined with mouthwash use were systematically evaluated.

The non-brushed groups were immersed in 20 mL of distilled water or one of two whitening mouthwashes for 6 and 12 h immediately after recording baseline measurements. The whitening mouthwashes were: (1) a formulation containing Patent Blue V (Colgate Optic White) and (2) a formulation containing activated charcoal (Colgate Plax White + Charcoal). The immersion durations were standardized at 6 h for the 6 month simulation group and 12 h for the 1 year simulation group, corresponding to daily 2 min mouthwash use over 6 months or 1 year, respectively. For the brushed groups, specimens underwent a brushing procedure (5 000 and/or 10 000 brushing cycles), followed by immersion in distilled water or one of the two whitening mouthwashes for 6 or 12 h, by the brushing duration.

Ra and VHN measurements were recorded at three different time points to assess changes in surface properties. Following the initial surface roughness measurements, a reference line was carefully marked on the composite resin disk surface to divide it into two halves, ensuring that the movement of the diamond indenter tip would not be obstructed during subsequent measurements. Subsequently, the initial microhardness measurements were conducted. The area where microhardness testing was performed was protected by applying adhesive tape to the composite disc.

**Fig. 1:**
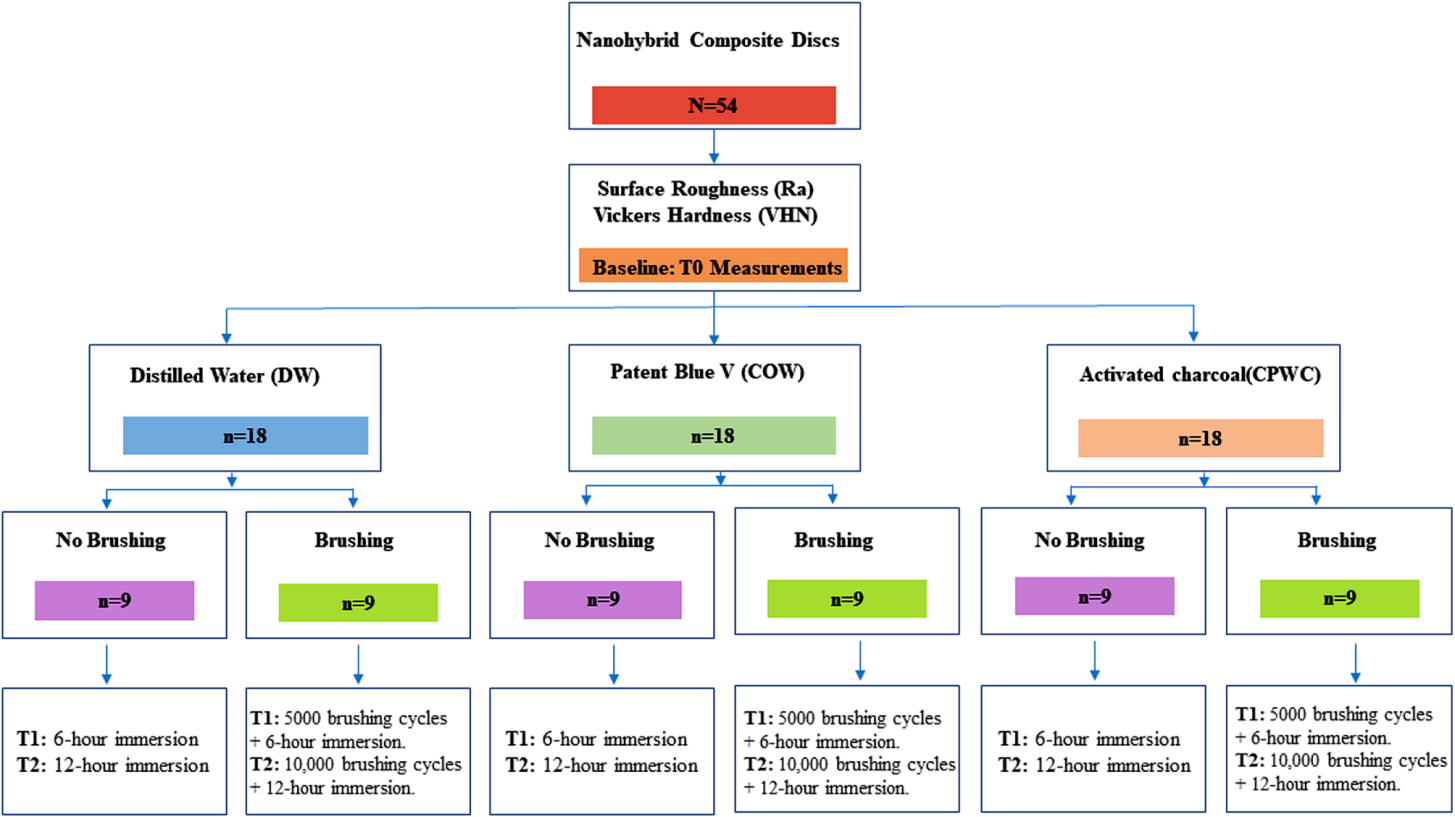
Schematic representation of study design

After this, the specimens were subjected to 5 000 brushing cycles using a standardized brushing machine. Following the brushing procedure, the same measurement protocols, including surface roughness and microhardness assessments, were repeated to evaluate the effects of abrasion. This process was continued, with the protected areas shielded by adhesive tape, up to 10 000 brushing cycles to observe the cumulative impact of prolonged mechanical stress. The tested groups in this study were as follows (Fig. 1):

#### Group 1

Non-Brushing + Distilled water.

#### Group 2

Non-Brushing + Patent Blue V-containing whitening mouthwashes.

#### Group 3

Non-Brushing + Charcoal-containing whitening mouthwashes.

#### Group 4

Brushing + Distilled water.

#### Group 5

Brushing + Patent Blue V-containing whitening mouthwashes.

#### Group 6

Brushing + Charcoal-containing whitening mouthwashes.

### Surface roughness analysis

Ra was evaluated using a contact mechanical profilometer (Surtronic S128, Taylor Hobson Ltd., Leicester, UK) with five µm diamond tips. The device, with a 4 mm measurement length and a 0.8 mm cutoff value, was positioned perpendicular to the brushing traces on the specimens. Before measurements, the device was calibrated using a standard plate system (Ra = 0.6 μm). Ra (µm) for each specimen was measured along three lines perpendicular to the brushing direction. Each specimen’s average surface roughness (ΔRa) was calculated by taking the mean of three measurements obtained from different points on the specimen.

### Surface microhardness analysis

The microhardness values of the composite surfaces were obtained using a microhardness tester (Shimadzu HMV/2L Ver 1.02, Shimadzu Corporation, Kyoto, Japan). The measurement area was initially localized at the lowest magnification (×10), followed by precise identification of the indentation site at ×40 magnification. A Vickers diamond indenter was applied with a load of 100 g for 15 s, creating indentations at three points on the designated test surface of each specimen. The diagonals of the indents were measured at ×40 magnification by aligning horizontal lines on the screen with their endpoints. The device automatically calculated the Vickers Hardness Number (VHN) based on the indent dimensions, including depth and edge length ratio. The average hardness value for each specimen was then calculated.

### Statistical analyses

A three-way mixed ANOVA was performed to evaluate the effects of experimental conditions (EC), time (T), and brushing condition (B), along with their interactions on Ra and VHN outcomes. To further analyze the data, one-way ANOVA followed by Bonferroni post hoc tests were conducted to compare ΔRa and ΔVHN at each time point and to assess differences among experimental conditions under both non-brushing and brushing scenarios. Additionally, repeated-measures one-way ANOVA with Bonferroni post hoc tests was used to examine time-dependent changes within each experimental condition (ΔRa and ΔVHN). Data normality was assessed using the Shapiro-Wilk test, while homogeneity of variance was verified with Levene’s test. Hypotheses were tested at a significance level of α = 0.05, and statistical analyses were conducted using IBM SPSS Statistics 26, with results expressed as mean ± standard deviation and a significance threshold of *p* < 0.05.

## Results

The three-way mixed ANOVA (Brushing × Experimental Conditions × Time) revealed significant effects on surface roughness (Ra) but no significant impact on microhardness (VHN). For surface roughness, significant main effects were found for experimental conditions (F(2, 48) = 5.752, *p* = 0.006) and time (F(2, 96) = 28.798, *p* < 0.001), indicating that whitening mouthwash type and immersion duration influenced surface roughness. Brushing showed a marginal but non-significant effect (F(1, 48) = 3.819, *p* = 0.056). Significant interactions were detected between brushing and time (F(2, 96) = 4.135, *p* = 0.019) and time and experimental conditions (F(4, 96) = 2.866, *p* = 0.027), while no significant interactions were found between brushing and experimental conditions (F(2, 48) = 0.894, *p* = 0.416) or for the three-way interaction (F(4, 96) = 0.996, *p* = 0.414) (Table 2). The means, standard deviations, and statistical analyses for surface roughness and microhardness average values are reported in Tables 3 and 4, respectively.

**Table 2:** Three-way mixed ANOVA results for surface roughness (Ra,µm) and Vickers microhardness (VHN)

Parameter	Source	Type III Sum of Squares	df	Mean square	F	*p*
Roughness	Brushing (B)	0.062	1	0.062	3819.000	0.056
	Experimental conditions (EC)	0.187	2	0.094	5.752	0.006 ^a^
	Time (T)	1.078	2	0.539	28.798	< 0.001^a^
	B x EC	0.029	2	0.015	0.894	0.416
	BxT	0.155	2	0.077	4.135	0.019 ^a^
	T x EC	0.215	4	0.054	2.866	0.027 ^a^
	B x T x EC	0.075	4	0.019	0.996	0.414
Microhardness	Brushing (B)	0.392	1	0.392	0.199	0.657
	Experimental conditions (EC)	5.644	2	2.822	1.433	0.249
	Time (T)	26.980	2	13.490	2.079	0.131
	B x EC	6.098	2	3.049	1.548	0.223
	BxT	5.106	2	2.553	0.393	0.676
	T x EC	19.095	4	4.774	0.736	0.570
	B x T x EC	34.658	4	8.665	1.335	0.262

EC: Experimental conditions; B: Brushing conditions; T: Time; BxEC: Brushing conditions versus Experimental conditions; TxEC: Time versus Experimental conditions; BxTxEC: Brushing conditions versus Time versus Experimental conditions.

^a^ Significant difference(*p* < 0.05)

**Table 3:** Mean(± standard deviation) of surface roughness (Ra,µm) according to experimental conditions (EC) and time (T) for each brushing condition (B)

Experimental Conditions	Time				
	T0	T1	T2		*p* ^b^
**Non Brushing**					
Control	0.917 (± 0.085) ^Aa^	0.930(± 0.059)^Aa^	0.932 (± 0.056)^Aa^		0.827
COW	0.937 (± 0.067) ^Aa^	0.974(± 0.192)^Aa^	0.992 (± 0.186)^Aa^		0.585
CPWC	0.947(± 0.47) ^Aa^	1.159 (± 0.315)^Aab^	1.243 (± 0.203)^Bb^		0.009 ^c^
*p* ^*a*^	0.641	0.077	0.001^c^		
**Brushing**					
Control	0.921(± 0.107)^Aa^	0.994(± 0.123)^Aa^	1.169(± 0.150)^Ab^		< 0.001 ^c^
COW	0.931(± 0.114)^Aa^	1.078(± 0.124)^Aab^	1.193(± 0.175)^Ab^		< 0.001 ^c^
CPWC	0.933(± 0.085)^Aa^	1.178(± 0.311)^Aa^	1.244(± 0.272)^Ab^		0.012 ^c^
*p* ^*a*^	0.964	0.188	0.732		

^a^ One-way ANOVA/Bonferroni

^b^ Repeated measures one-way ANOVA/Bonferroni

^c^ Statistically significant difference (*p* < 0.05). For each evaluation time, means with different superscript uppercase (in the columns) and lowercase (in the rows) letters indicate a statistically significant difference (*p* < 0.05)

**Table 4:** Mean (± standard deviation) of Vickers microhardness (VHN) according to experimental conditions (EC) and time (T) for each brushing condition (B)

Experimental Conditions	Time			
	T0	T1	T2	*p* ^b^
**Non Brushing**				
Control	40.3 (± 2.5)^Aa^	42.3(± 2.7)^Aa^	41.1 (± 2.5)^Aa^	0.282
COW	39.4 (± 2.7)^Aa^	39.5(± 1.9)^Ba^	40.7 (± 2.8)^Aa^	0.391
CPWC	41.4(± 2.3)^Aa^	41.5(± 2.2)^Aa^	40.0 (± 2.9)^Aa^	0.454
*p* ^*a*^	0.271	0.042	0.682	
**Brushing**				
Control	40.0(± 1.8)^Aa^	42.2(± 2.3)^Aa^	41.2(± 2.8)^Aa^	0.150
COW	40.8(± 3.1)^Aa^	41.4(± 2.5)^Aa^	40.7(± 2.3)^Aa^	0.842
CPWC	39.4(± 2.5)^Aa^	40.4(± 2.8)^Ab^	41.5(± 2.2)^Ab^	0.296
*p* ^*a*^	0.484	0.328	0.756	

^a^ One-way ANOVA/Bonferroni

^b^ Repeated measures one-way ANOVA/Bonferroni

^c^ Statistically significant difference (*p* < 0.05). For each evaluation time, means with different superscript uppercase (in the columns) and lowercase (in the rows) letters indicate a statistically significant difference (*p* < 0.05)

### Surface roughness alterations (ΔRa)

Table 3 summarizes the mean surface roughness values (Ra, µm) for each experimental condition at different time points.

### Non-brushing groups

In the Control and COW groups, surface roughness remained relatively stable over time, with non-significant increases of + 0.015 μm (1.6%) and + 0.055 μm (5.9%), respectively (*p* = 0.827 and *p* = 0.585). In contrast, the CPWC group showed a statistically significant increase of + 0.296 μm (31.2%) at T2 compared to baseline (*p* = 0.009).

At T2, CPWC exhibited the highest surface roughness in the non-brushed condition (1.243 ± 0.203 μm), which was significantly greater than both Control (0.932 ± 0.056 μm, *p* = 0.001) and COW (0.992 ± 0.186 μm, *p* = 0.001). The relative increase in CPWC was 29.6% higher than Control and 25.3% higher than COW.

### Brushing groups

In all brushed groups, surface roughness increased significantly over time (*p* < 0.05). In the Control group, Ra increased by + 0.248 μm (26.9%) from baseline (0.921 ± 0.107 μm) to T2 (1.169 ± 0.150 μm, *p* < 0.001). In the COW group, the increase was + 0.262 μm (28.1%) (*p* < 0.001). The CPWC brushing group showed the highest growth, with + 0.311 μm (33.3%) from 0.933 ± 0.085 μm to 1.244 ± 0.272 μm at T2 (*p* = 0.012).

When comparing brushing versus non-brushing, surface roughness at T2 in the CPWC brushing group (1.244 ± 0.272 μm) was similar to that in the non-brushing group (1.243 ± 0.203 μm), indicating that brushing did not significantly amplify roughness beyond the effect of the mouthwash alone (*p* > 0.05). In contrast, the Control and COW groups exhibited higher Ra values under brushing conditions than their respective non-brushing counterparts, although these differences were not statistically significant (*p* > 0.05).

### Vickers microhardness alterations (ΔVHN)

The mean microhardness values (VHN) across all groups and time points are provided in Table 4.

### Non-brushing groups

Microhardness remained stable over time in the Control (*p* = 0.282) and CPWC (*p* = 0.454) groups, showing slight changes of + 0.8 VHN (+ 1.98%) and − 1.4 VHN (− 3.38%), respectively. The COW group exhibited a transient reduction at T1 (− 0.9 VHN, − 2.5%) and a recovery at T2 (+ 1.3 VHN, + 3.3%, *p* = 0.391).

At T2, CPWC exhibited the most significant reduction in VHN (− 3.38%), which was significantly lower than both Control (+ 1.98%) and COW (+ 3.3%, *p* = 0.001). The difference between CPWC and COW was 1.72% (− 0.7 VHN).

### Brushing groups

No statistically significant changes in microhardness were observed over time in any of the brushed groups (*p* > 0.05). The Control group showed a non-significant increase of + 2.2 VHN (5.5%) (*p* = 0.150), and the COW group remained virtually unchanged (− 0.1 VHN, − 0.2%, *p* = 0.842). The CPWC brushing group exhibited a slight initial decrease at T1 (− 1.0 VHN, − 2.5%) followed by an increase at T2 (+ 2.1 VHN, + 5.33%, *p* = 0.756).

At T2, VHN values were comparable between brushing and non-brushing conditions in all groups, indicating that brushing did not significantly influence microhardness over time (*p* > 0.05).

## Discussion

The increasing demand for esthetic dental treatments has led to the widespread use of whitening mouthwashes containing Patent Blue V and activated charcoal. Yet, their effects on restorative materials remain largely underexplored. This study evaluated the impact of Patent Blue V- and activated charcoal-containing whitening mouthwashes (COW and CPWC) on the surface roughness (Ra), and Vickers microhardness (VHN) of a nanohybrid resin composite under both brushed and non-brushed conditions. The null hypothesis, which proposed that these mouthwashes would not significantly affect Ra or VHN, was partially rejected. The findings demonstrated that CPWC significantly increased Ra, particularly in both brushed and non-brushed groups, whereas no significant alterations in VHN were observed across all conditions.

Assessing the effects of prophylactic measures, such as brushing, mouthwash use, or their combination, on restorative materials’ surface and physical properties is crucial for maintaining restoration integrity. Studies suggest that tooth whitening can impact natural teeth and the microhardness, surface roughness, and texture of resin composite restorations [24, 25, 26, 27, 28].

The Ra analysis demonstrated that brushing contributed to an overall increase in surface roughness across all experimental groups. However, the differences between brushed and non-brushed subgroups were relatively small, suggesting that mechanical action alone was not the primary factor in surface roughness progression. Specifically, at T2, ΔRa increased by 26.9% in Control, 28.1% in COW, and 33.3% in CPWC for brushed groups, while the corresponding increases in non-brushed groups were 1.6%, 5.9%, and 31.2%, respectively. These findings indicate that the rise in Ra was not solely due to mechanical abrasion but also influenced by chemical interactions with the whitening mouthwash solutions. The highest increase in Ra was observed in CPWC under both conditions, suggesting a synergistic effect between the abrasiveness of activated charcoal and the mechanical effects of brushing. While brushing alone contributed to surface roughness in all groups, the pronounced progression observed in CPWC indicates that abrasive particles suspended in the solution may play a dominant role.

Dionysopoulos et al. [28] previously reported that charcoal-based whitening toothpaste significantly altered enamel surface morphology, increasing roughness and creating crater-like structures. However, no significant effect was observed when charcoal-based mouthwash was combined with brushing, similar to Sultan et al. [27] Similarly, Sultan et al. [23] found that the control group exhibited the highest mean Ra value, followed by the OW group, while the CP group showed the lowest mean Ra value, indicating that whitening mouthwashes influenced surface roughness differently depending on their formulation.

In contrast, the present study demonstrated that CPWC significantly increased Ra in nanohybrid composites even in the absence of brushing, highlighting the material-specific nature of whitening mouthwash effects. The distinct chemical and mechanical responses of resin composites and enamel may explain these differences. These findings suggest that while activated charcoal may not inherently alter enamel roughness, its prolonged exposure, particularly in combination with brushing, may contribute to surface degradation in resin-based materials. Given the potential impact of increased surface roughness on plaque accumulation and stain susceptibility, careful evaluation is necessary when recommending charcoal-containing mouthwashes for patients with composite restorations.

The significant increase in Ra, particularly in CPWC groups, may pose esthetic and functional challenges by promoting plaque accumulation, bacterial adhesion, and staining. Ra values exceeding the clinically acceptable 0.2 μm threshold can enhance biofilm formation and reduce wear resistance [6, 7, 8]. In this study, all groups surpassed this threshold, likely due to the combined effects of brushing, whitening mouthwash exposure, and the limitations of the polishing process. Although the Sof-Lex system is known for achieving optimal smoothness, previous studies indicate that no composite material reaches the 0.2 μm threshold after polishing [8, 29, 30]. Mechanical brushing contributed to surface wear, while the chemical nature of CPWC may have altered the organic matrix of the composite, further increasing Ra. These findings highlight the need to carefully select whitening mouthwashes, particularly for patients with composite restorations, as external factors such as brushing force, application frequency, and mouthwash composition can significantly impact surface properties and long-term clinical durability.

The between-group comparisons at T2 confirmed that CPWC had the highest Ra increase in the non-brushed condition, with a 29.6% higher value than Control and 25.3% higher than COW (*p* = 0.001). In the brushed condition, CPWC still exhibited the most significant Ra increase, 6.4% higher than Control and 4.3% higher than COW, although these differences were not statistically significant. These results suggest that the abrasiveness of activated charcoal played a more prominent role in surface roughness progression than mechanical brushing alone. Furthermore, the progressive increase in Ra from T0 to T2 in CPWC groups underscores the importance of exposure duration as a critical factor in surface degradation. This suggests that even short-term use of charcoal-containing products may lead to early surface deterioration on resin composites. These results agree with previous studies that identified mechanical abrasion as a major contributor to surface wear and roughness changes [31, 32]. The findings emphasize the universal impact of brushing on surface degradation while suggesting that chemical interactions—particularly those involving abrasive agents—may exacerbate these effects.

Several studies have reported that mouthwashes, particularly those with low pH and alcohol, can degrade the resin matrix, leading to reductions in microhardness and increased surface roughness [1, 13, 14, 15, 16, 17, 18]. However, in this study, no statistically significant changes in VHN were observed, likely due to the neutral to slightly alkaline pH of the tested mouthwashes (COW: 7.0; CPWC: 7.9). Under non-brushed conditions, CPWC exhibited a minor but statistically significant decrease in VHN (–1.4 VHN, − 3.38%), which was lower than both Control (+ 0.8 VHN, + 1.98%) and COW (+ 1.3 VHN, + 3.3%). This reduction may be attributed to the interaction between activated charcoal and the polymer matrix, as the adsorptive nature of charcoal could influence surface properties over time.

Conversely, in the brushed condition, CPWC demonstrated a non-significant increase in microhardness (+ 2.1 VHN, + 5.33%), reaching the highest recorded VHN value at T2 (41.5 ± 2.2). This trend toward increased surface hardness may be related to the mechanical polishing effect of brushing, which could remove the outermost resin layer and expose more densely cross-linked or filler-rich regions. Alternatively, brushing might compact the surface, increasing localized hardness. Despite the abrasive potential of charcoal, the composite’s overall microhardness remained stable, suggesting that its internal mechanical structure was preserved. These contrasting results align with prior findings that immersion in distilled water or neutral solutions may lead to slight increases in microhardness due to continued polymer cross-linking and water absorption [13, 33].

Hamdy et al. [1] reported a significant reduction in nanohybrid resin composites’ microhardness following exposure to whitening mouthwashes, including Chlorhexidine, Listerine Green Tea, and Colgate Optic White. In contrast, the present study did not observe any statistically significant decrease in VHN, suggesting that the tested composite material demonstrated higher resistance to chemical degradation under the evaluated conditions. This discrepancy may be attributed to compositional differences among resin composites, particularly in filler type, content, and polymerization dynamics, which influence a material’s chemical stability and mechanical response.

The findings of the current investigation underscore that although whitening mouthwashes—particularly those containing activated charcoal—significantly influence surface roughness, their impact on microhardness remains minimal. Nevertheless, the mechanical and chemical behavior of resin composites highly depends on their formulation. In this study, Clearfil Majesty™ Esthetic, a widely used multi-shade nanohybrid composite designed for anterior restorations, was selected owing to its high mechanical strength, esthetic potential, and broad clinical applicability [34, 35]. While using this well-characterized material enhances the comparability of results with existing literature, it also constitutes a methodological limitation, as findings derived from a single formulation cannot be generalized to all composite types. Resin composites with differing filler particle morphology, size distribution, and polymer matrices may exhibit divergent responses to chemical or mechanical challenges posed by whitening agents.

To date, no prior studies have systematically investigated the effects of mouthwashes containing Patent Blue V or activated charcoal on the surface properties of resin composites. This novelty restricts direct comparisons but simultaneously highlights the relevance of the present findings. However, certain methodological constraints should be acknowledged when interpreting the results. The in vitro nature of the study does not fully replicate the complex dynamics of the oral environment, where variables such as salivary flow, enzymatic activity, thermal fluctuations, and biofilm formation significantly influence the aging and degradation of restorative materials. Although the brushing simulation employed a standardized load and motion pattern, it did not account for interindividual variations in brushing frequency, force, and technique. Furthermore, the immersion model used for mouthwash exposure may not entirely reflect clinical usage patterns, in which exposure times and rinsing behaviors are more transient and varied.

Future research should address these limitations by incorporating multiple resin composite formulations with varying filler technologies and polymer matrices and simulating more clinically relevant conditions, such as thermocycling, pH cycling, and biofilm activity. Longitudinal in vivo or situ designs would provide greater insight into the cumulative effects of whitening mouthwashes on restorative materials and allow evaluation of additional outcomes, such as color stability, wear resistance, and bacterial adhesion.

Despite these limitations, the current study provides valuable preliminary evidence regarding the influence of activated charcoal-containing mouthwashes on nanohybrid resin composites. The methodological rigor employed—including controlled brushing simulation, standardized polishing, and repeated measurements—enhances the internal validity of the findings. These results contribute to the evolving knowledge that informs clinical decision-making, particularly in selecting oral hygiene products that preserve resin-based restorations’ esthetic and functional integrity.

From a clinical perspective, future studies should aim to develop evidence-based oral hygiene protocols that optimize whitening efficacy while minimizing surface degradation. Investigations focusing on mitigating surface roughness, enhancing composite resilience, and understanding the long-term interaction between whitening agents and restorative materials will aid in formulating recommendations that sustain both esthetic outcomes and material longevity.

## Conclusion

This study demonstrated that whitening mouthwashes, particularly those formulated with activated charcoal, significantly increased the surface roughness of nanohybrid resin composite, with the most pronounced alterations observed in the CPWC groups. Brushing further exacerbated this effect, indicating a synergistic interaction between chemical and mechanical degradation processes. In contrast, no statistically significant changes were detected in microhardness, suggesting that the composite’s internal mechanical integrity was not compromised despite surface-level deterioration.

From a clinical standpoint, these findings underscore the potential risks associated with the routine use of activated charcoal-containing mouthwashes in patients with composite restorations. Although microhardness remained stable, the substantial increase in surface roughness may predispose restorations to plaque accumulation, staining, and premature wear. Therefore, careful consideration should be given when recommending whitening mouthwashes, with emphasis placed on selecting formulations that preserve both the esthetic and functional longevity of restorative materials.

## Data Availability

The datasets used and/or analysed during the current study are available from the corresponding author on reasonable request.

## Abbreviations

ANOVA: Analysis of Variance
B: Brushing Condition
COW: Patent Blue V-containing Whitening Mouthwash
CPWC: Charcoal-containing Whitening Mouthwash
DW: Distilled Water
EC: Experimental Conditions
LED: Light-Emitting Diode
OTC: Over-the-Counter
Ra: Surface Roughness
RPM: Revolutions Per Minute
SiC: Silicon Carbide
SPSS: Statistical Package for the Social Sciences
T: Time
T0 / T1 / T2: Time points (Baseline, After 6 h, After 12 h)
VHN: Vickers Hardness Number

## References

[1] Hamdy TM, Abdelnabi A, Othman MS, Bayoumi RE, Abdelraouf RM. Effect of different mouthwashes on the surface microhardness and color stability of dental nanohybrid resin composite. Polym (Basel). 2023;15(4):815. 10.3390/polym15040815 PMC9961015 36850099

[2] Elmalawany LM, El-Refai DA, Alian GA. Change in surface properties of two different dental resin composites after using various beverages and brushing. BMC Oral Health. 2023;23(1):966. 38053124 10.1186/s12903-023-03710-8 PMC10696683

[3] Nasoohi N, Hadian M, Hoorizad M, Hashemi SS, Naziri Saeed SH. In-vitro effect of alcohol and non-alcohol mouthwash on color change of two types of bleach shade composite. J Res Dent Maxillofac Sci. 2019;4(2):1–6.

[4] Ghajari MF, Shamsaei M, Basandeh K, Galouyak MS. Abrasiveness and whitening effect of charcoal-containing whitening toothpastes in permanent teeth. Dent Res J (Isfahan). 2021;18:51. 34497686 PMC8404563

[5] Epple M, Meyer F, Enax J. A critical review of modern concepts for teeth whitening. Dent J. 2019;7(3):79. 10.3390/dj7030079 PMC6784469 31374877

[6] O’Neill C, Kreplak L, Rueggeberg FA, Labrie D, Shimokawa CAK, Price RB. Effect of tooth brushing on gloss retention and surface roughness of five bulk-fill resin composites. J Esthet Restor Dent. 2018;30(1):59–69. 29205770 10.1111/jerd.12350

[7] Yilmaz C, Kanik O. Investigation of surface roughness values of various restorative materials after brushing with blue covarine containing whitening toothpaste by two different methods: AFM and profilometer. Microsc Res Tech. 2022;85(2):521–32. 34528740 10.1002/jemt.23925

[8] Alharbi G, Al Nahedh HN, Al-Saud LM, Shono N, Maawadh A. Effect of different finishing and Polishing systems on surface properties of universal single shade resin-based composites. BMC Oral Health. 2024;24(1):197. 38326838 10.1186/s12903-024-03958-8 PMC10848531

[9] Jamwal N, Rao A, Mc GS, K RS, Bh MP, Jodalli P, Ks A, Br A. Effect of whitening toothpastes on the surface roughness and microhardness of human teeth-an in vitro study. Clin Oral Investig. 2023;27(12):7889–97. 37966513 10.1007/s00784-023-05381-9 PMC10713793

[10] Islam MS, Padmanabhan V, Shanati KA, Naser AM, Hashim NT, Aryal ACS. Comparative analysis of whitening outcomes of over-the-counter toothpastes: an in vitro study. Dent J (Basel). 2025;13(2):45. 39996919 10.3390/dj13020045 PMC11854843

[11] Barbosa LMM, Amancio Filha MBG, Leite JVC, Santos J, De Medeiros JM, De Oliveira ILM, Pecho OE, Meireles SS, Lima RBW. Over-the-counter products in tooth bleaching: A scoping review. J Dent. 2024;145:104989. 38582435 10.1016/j.jdent.2024.104989

[12] Ribeiro EP, Zanin GT, Goncalves AE, Kury M, Cavalli V, Guiraldo RD, Lopes MB, Berger SB. Whitening efficacy of activated charcoal-based products: A single-blind randomized controlled clinical trial. J Dent. 2024;143:104877. 38316199 10.1016/j.jdent.2024.104877

[13] Al-Saud LM, Alolyet LM, Alenezi DS. The effects of selected mouthwashes on the surface microhardness of a single-shade universal resin composite: in vitro study. J Adv Oral Res. 2022;13(2):234–44.

[14] Manzoor S, Arooj Z, Waqas MA, Irshad N, Saeed A, Malik A, Sarfaraz Z, Shaukat MS. Surface microhardness of microhybrid and nanocomposite after storage in mouth washes. J Ayub Med Coll Abbottabad. 2022;34(3):540–7. 36377172 10.55519/JAMC-03-10181

[15] da Silva EM, de Sa Rodrigues CU, Dias DA, da Silva S, Amaral CM, Guimaraes JG. Effect of toothbrushing-mouthrinse-cycling on surface roughness and topography of nanofilled, microfilled, and microhybrid resin composites. Oper Dent. 2014;39(5):521–9. 24304341 10.2341/13-199-L

[16] Gehlot PM, Sudeep P, Manjunath V, Annapoorna BM, Prasada LK, Nandlal B. Influence of various desensitizing mouthrinses and simulated toothbrushing on surface roughness and microhardness of tetric n-ceram bulk-fill resin composite: an in vitro study and scanning electron microscope analysis. Eur J Dent. 2022;16(4):820–7. 35176786 10.1055/s-0041-1739547 PMC9683869

[17] Mara da Silva T, Barbosa Dantas DC, Franco TT, Franco LT. Rocha Lima Huhtala MF. Surface degradation of composite resins under staining and brushing challenges. J Dent Sci. 2019;14(1):87–92. 30988884 10.1016/j.jds.2018.11.005 PMC6445979

[18] Trauth KG, Godoi AP, Colucci V, Corona SA, Catirse AB. The influence of mouthrinses and simulated toothbrushing on the surface roughness of a nanofilled composite resin. Braz Oral Res. 2012;26(3):209–14. 22641439 10.1590/s1806-83242012000300005

[19] Vieira-Junior WF, Lima DA, Tabchoury CP, Ambrosano GM, Aguiar FH, Lovadino JR. Effect of toothpaste application prior to dental bleaching on whitening effectiveness and enamel properties. Oper Dent. 2016;41(1):E29–38. 26449589 10.2341/15-042-L

[20] Vaz VTP, Jubilato DP, Oliveira MRM, Bortolatto JF, Floros MC, Dantas AAR, Oliveira Junior OB. Whitening toothpaste containing activated charcoal, blue covarine, hydrogen peroxide or microbeads: which one is the most effective? J Appl Oral Sci. 2019;27:e20180051. 30673027 10.1590/1678-7757-2018-0051 PMC6438662

[21] Faul F, Erdfelder E, Lang AG, Buchner A. G*Power 3: a flexible statistical power analysis program for the social, behavioral, and biomedical sciences. Behav Res Methods. 2007;39(2):175–91. 17695343 10.3758/bf03193146

[22] ISO 4049:2019. Dentistry-Polymer-based restorative materials. international organization for standardization: Geneva, Switzerland.

[23] Ntovas P, Masouras K, Lagouvardos P. Efficacy of non-hydrogen peroxide mouthrinses on tooth whitening: an in vitro study. J Esthet Restor Dent. 2021;33(7):1059–65. 34228393 10.1111/jerd.12800

[24] Polydorou O, Hellwig E, Auschill TM. The effect of different bleaching agents on the surface texture of restorative materials. Oper Dent. 2006;31(4):473–80. 16924988 10.2341/05-75

[25] Polydorou O, Monting JS, Hellwig E, Auschill TM. Effect of in-office tooth bleaching on the microhardness of six dental esthetic restorative materials. Dent Mater. 2007;23(2):153–8. 16472855 10.1016/j.dental.2006.01.004

[26] Polydorou O, Hellwig E, Auschill TM. The effect of at-home bleaching on the microhardness of six esthetic restorative materials. J Am Dent Assoc. 2007;138(7):978–84. quiz 1022. 17606497 10.14219/jada.archive.2007.0295

[27] Sultan MS. Effect of hydrogen peroxide versus charcoal-based whitening mouthwashes on color, surface roughness, and color stability of enamel. BMC Oral Health. 2024;24(1):897. 39107715 10.1186/s12903-024-04631-w PMC11302163

[28] Dionysopoulos D, Papageorgiou S, Malletzidou L, Gerasimidou O, Tolidis K. Effect of novel charcoal-containing whitening toothpaste and mouthwash on color change and surface morphology of enamel. J Conserv Dent. 2020;23(6):624–31. 34083921 10.4103/JCD.JCD_570_20 PMC8095700

[29] Zhang L, Yu P, Wang XY. Surface roughness and gloss of polished nanofilled and nanohybrid resin composites. J Dent Sci. 2021;16(4):1198–203. 34484588 10.1016/j.jds.2021.03.003 PMC8403785

[30] Soliman HAN, Elkholany NR, Hamama HH, El-Sharkawy FM, Mahmoud SH, Comisi JC. Effect of different Polishing systems on the surface roughness and gloss of novel nanohybrid resin composites. Eur J Dent. 2021;15(2):259–65. 33111284 10.1055/s-0040-1718477 PMC8184273

[31] AlAli M, Silikas N, Satterthwaite J. The effects of toothbrush wear on the surface roughness and gloss of resin composites with various types of matrices. Dent J. 2021;9(1). 10.3390/dj9010008 PMC7827053 33445457

[32] Hojo FR, Martins TC, Vieira-Junior WF, Franca F, Turssi CP, Basting RT. Coating agents for resin composites: effect on color stability, roughness, and surface micromorphology subjected to brushing wear. Oper Dent. 2025;50(1):101–14. 39763325 10.2341/24-069-L

[33] Diab M, Zaazou M, Mubarak E, Olaa M. Effect of five commercial mouthrinses on the microhardness and color stability of two resin composite restorative materials. J Aust J Basic Appl Sci. 2007;1(4):667–74.

[34] Celik N, Iscan Yapar M. Colour stability of stained composite resins after brushing with whitening toothpaste. Int J Dent Hyg. 2021;19(4):413–20. 34101339 10.1111/idh.12529

[35] Baltacioglu IH, Demirel G, Kolsuz ME, Orhan K. The effect of gravity on marginal integrity of different flowable bulk-fill resin composites. Med (Kaunas Lithuania). 2024;60(3). 10.3390/medicina60030396 PMC10971810 38541122

